# Determination of mesopores in the wood cell wall at dry and wet state

**DOI:** 10.1038/s41598-020-65066-1

**Published:** 2020-06-12

**Authors:** Martin Nopens, Uta Sazama, Sandra König, Sergej Kaschuro, Andreas Krause, Michael Fröba

**Affiliations:** 1 Universität Hamburg, Department Biology, Institute of Wood Science, Wood Physics, Leuschnerstraße 91 c, 21031 Hamburg, Germany; 2 Universität Hamburg, Department Chemistry, Institute of Inorganic and Applied Chemistry, Martin-Luther-King-Platz 6, 20146 Hamburg, Germany; 3 Thünen Institute, Institute of Wood Research, Leuschnerstr 91, 21031 Hamburg, Germany

**Keywords:** Structural biology, Biomaterials, Nanoscale materials

## Abstract

Wood porosity is of great interest for basic research and applications. One aspect is the cell wall porosity at total dry state. When water is absorbed by wood, the uptake of water within the cell wall leads to a dimension change of the material. A hypothesis for possible structures that hold the water is induced cell wall porosity. Nitrogen and krypton physisorption as well as high pressure hydrogen sorption and thermoporosimetry were applied to softwood and hardwood (pine and beech) in dry and wet state for determining surface area and porosity. Physisorption is not able to detect pores or surface area within the cell wall. Krypton physisorption shows surface area up 5 times lower than nitrogen with higher accuracy. With high pressure sorption no inaccessible pore volumes were seen at higher pressures. Thermoporosimetry was not able to detect mesopores within the hygroscopic water sorption region. Physisorption has to be handled carefully regarding the differences between adsorptives. The absence of water-induced mesopores within the hygroscopic region raise doubts on existing water sorption theories that assume these pore dimensions. When using the term “cell wall porosity”, it is important to distinguish between pores on the cell wall surface and pores that exist because of biological structure, as there are no water-induced mesopores present. The finding offers the possibility to renew wood-water-sorption theories because based on the presented results transport of water in the cell wall must be realized by structures lower than two 2 nm. Nanoporous structures in wood at wet state should be investigated more intensively in future.

## Introduction

Wood is used for a wide range of applications like furniture, construction components or composites. To increase the usage of wood in products with higher technical requirements (reinforced or functionalized materials), understanding of the basic structure is crucial. Water governs the properties as well as the structure of wood at different climate conditions. Free water molecules from the ambient atmosphere try to gain equilibrium with water molecules in wood cell wall. In the range between 0 and close to 100% relative humidity, water absorption and desorption takes place, leading to different equilibrium moisture contents. The water sorption in this hygroscopic region leads to a deformation of the cell wall, commonly called swelling and shrinking. Therefore the term absorption is used^1^. At a certain level, which is individual for each species, saturation of the wooden cell wall with water is reached. Above this so-called fiber saturation point added water remains outside the cell wall, either in the cell lumina or in other biological structures like pits.

Wood cell walls contain cellulose, hemicellulose, lignin and various other organic and inorganic molecules, which are characteristic for each species. Despite the knowledge of these compounds, less information is available on the structural organization of all components on molecular level.

Hydroxyl groups of cellulose, hemicellulose and lignin are the main contributors for water sorption^2,3^. When water is absorbed into wood, a specific space is needed. This leads to the question where the water is held within the wooden structure. This issue has been part of intensive research for decades^4,5^. Water in cellulose, for example, has been classified as free water, freezing-bound water and non-freezing bound water^6^. However, for wood it is assumed that only non-freezing bound water is present in the cell wall^5,7^. One assumption could be that water forms kind of porosity within the cell wall when absorption takes place.

Due to the sigmoidal shape of wood sorption isotherms, several explanations have been made in the past to distinguish water states. For example mono- and multilayer models (BET, Langmuir or Hailwood Horrobin) as well as cluster theories^8–10^.

The structure of wood components as well as the understanding of water bonding at different moisture contents is important for a wide range of properties and applications, for example chemical modification, decay by fungi or technical properties like creep. One hypothesis for wood swelling is the appearance of water induced porosity within the cell wall^11,12^. It is important to differentiate between pores in the dry and wet state. Also a distinction is needed to differentiate between biological structures which could be detected as pores and ones evoked by water vapor sorption.

Pores can be classified into micropores (<2 nm), mesopores (2–50 nm) and macropores which are above 50 nm as it is done by the IUPAC system^13^. Different pore shapes like slit, cylindrical, spherical pores or for example, a highly interconnected sponge like pore system is observed for different materials.

Different experimental methods are applicable for porosity investigations. A summary of several methods for pore-detection in wood is found in Hill^14^. To evaluate the density and porosity of the cell wall gas pycnometry is used^12,15^. Porosity in the mesoporous and macroporous range can be detected with mercury intrusion porosimetry^15,16^. Pore sizes of the wood are expected to be in the mesoporous range^17–20^ and also in the microporous range^21^. For these pore sizes, gas physisorption and cryoporosimetry are the most important methods to use.

Nitrogen physisorption is commonly used for the determination of pore size distribution and surface area of highly porous non swelling materials^22^. The technique has also been applied to pulp and cellulose where the extracted lignin and hemicellulose left space for gaseous molecules to adsorb^23–25^. It has been reported that the cell wall of wood in its completely dry state is non-porous with a surface area of 1 m²/g^26^. Nitrogen physisorption was used for the determination of wood porosity and surface area also in newer studies^18,27–29^.

Another species of gas which can be used to detect small surface areas of inorganic materials and thin films is krypton^30–32^. Krypton has also been applied for organic materials like cellulose^25,33^.

Gas physisorption can only detect accessible pores^34^. Within the dry wood cell wall, areas of non-accessible parts can exist without any contact to the outer environment. High pressure hydrogen sorption might overcome this limitation and detect pores in blocked areas in the cell wall which open at higher gas pressures as it is done for metal-organic frameworks^35^.

The pore size influences the freezing and melting behavior in meso- and macropores. With smaller pore size, freezing temperature is shifted to lower temperatures (Table 1). This behavior can be investigated by cryoporometry.

**Table 1:** Isothermal step heating program with related pore size distribution based on the Gibbs-Thomsen-Equation for cylindrical pores^47^, heating rate 1 K/min.

Step	Temperature °C	Pore diameter (nm)
1	−10	4
2	−6	6.6
3	−4	9.9
4	−2	19.8
5	−1.5	26.4
6	−1.1	36
7	−0.8	49.5
8	−0.5	79.2
9	−0.2	198

Assuming water in a cylindrical pore, the presence of a non-freezing zone adjoining to the pore wall surface has been proven^36^. Therefore, this zone has to be taken into account when determining pore sizes with cryoporometry. One limitation of the method in general is the resolution boundary of 2–3 nm pore size. Below this pore size no melting of water can be observed^37–39^.

Another method using the melting depression for pore size determination is the application of NMR-cryoporometry. This has been applied to polymers, pulp and native wood^20,40,41^. Another approach is the use of differential scanning calorimetry (DSC). This method is broadly available and data evaluation is easier than with other methods. Thermoporosimetry (TPM) is widely used to detect pores in inorganic materials like MCM-41 silicas and others^37,42–46^. It has also been applied to organic materials like pulp and cellulose^25,45,47–52^. Some studies can also be found for native wood^53^, dried wood^19^, and modified wood^54,55^. All these investigations were done exclusively in the fully saturated state.

A linear heating rate is often used to detect different melting zones and therefore pore sizes as well as the corresponding amounts^25,42,46^. Due to the problem that the melting temperatures are influenced by the heating rate, an isothermal step procedure with holding times at different temperatures can be used^45^. When calculating the pore size by the amount of melted water with the enthalpy of the isothermal step procedure, it is important to take into account the sensible heat^55^. This was done for wood only for some investigations^45,53,55^. It was previously assumed that a moisture content above fiber saturation is needed for TPM in wood^54^, but until now no separation between preexisting and water induced pores has been made.

The aim of this study is to clarify if mesopores can be detected in the wood cell wall at dry and wet state. Additionally, we want to ascertain if pores are opened during the water sorption process. We have used physisorption to investigate wood cell wall surface area in the dry state by with nitrogen and krypton. High pressure hydrogen physisorption was applied to dry and wet wood samples to discover possible pore blocking. TPM and DSC measurements have been used to clarify if water-induced cell wall meso-porosity exists within the hygroscopic region.

## Results and Discussion

### Gas physisorption

**Figure 1:**
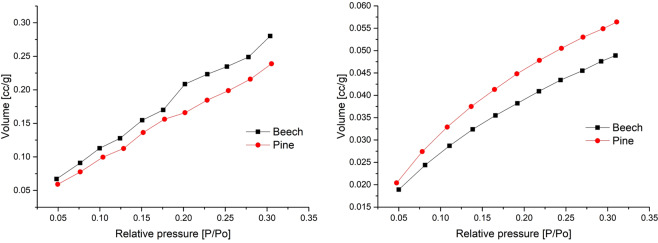
Example of Nitrogen (left) and Krypton (right) physisorption isotherms for beech and pine.

Physisorption isotherms of nitrogen and krypton on beech and pine wood can be seen in Fig. 1. The resulting BET surface areas are shown in Table 2. Nitrogen physisorption measurements show surface areas between 1 and 2 m²/g, whereas krypton physisorption measurements exhibit surface areas between 0.2 and 0.3 m²/g. The standard deviation is 10–100 times lower for krypton than for nitrogen.

**Table 2:** Nitrogen physisorption of pine and beech with correlation coefficient and mean and standard deviation values for several measurements (left nitrogen, right krypton).

Nitrogen			Krypton		
pine, sapwood, n = 6	BET surface area [m²/g]	r	pine, sapwood, n = 6	BET surface area [m²/g]	r
mean	1,533	0,8973	mean	0,251	0,9994
standard deviation	0,619	0,0726	standard deviation	0,008	0,0002
beech, sapwood n = 6			beech, sapwood n = 6		
mean	1,116	0,9602	mean	0,205	0,9994
standard deviation	0,392	0,0307	standard deviation	0,012	0,0003

**Table 3:** Krypton physisorption on douglas fir, heartwood and sapwood, latewood and earlywood, with correlation coefficient and mean and standard deviation values for several measurements.

Douglas fir, heartwood, latewood, n = 3	BET surface area [m²/g]	r
Mean	0,144	0,9990
standard deviation	0,008	0,0002
Douglas fir, heartwood, earlywood, n = 3	BET surface area [m²/g]	r
Mean	0,432	0,9988
standard deviation	0,016	0,0006
Douglas fir, sapwood, latewood, n = 3	BET surface area [m²/g]	r
Mean	0,057	0,9965
standard deviation	0,010	0,0022
Douglas fir, sapwood, earlywood, n = 3	BET surface area [m²/g]	r
Mean	0,361	0,9989
standard deviation	0,007	0,0001

**Figure 2:**
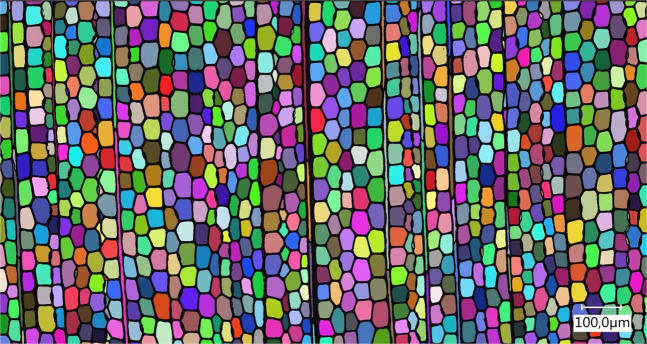
Example of pore lumina detection by microscopic measurements of douglas fir sapwood (earlywood).

Microscopic cell lumina detection was performed for Douglas fir sapwood (Fig. 2) to detect individual surface areas and circumferences. The surface areas from microscopic measurements can be seen in Table 4. Results of both methods (Tables 3 and 4) show comparable values.

**Table 4:** Surface area of earlywood and latewood from Douglas fir sapwood (microscopic pore area measurements).

Douglas fir, sapwood, latewood, n = 3	microscopic surface area [m²/g]
Mean	0,068
standard deviation	0,001
Douglas fir, sapwood, earlywood, n = 3	microscopic surface area [m²/g]
Mean	0,261
standard deviation	0,041

The experimentally determined values of surface area for nitrogen (Table 2) correspond well with the literature^17,27,56^. However, the standard deviation based on repeated measurements is about one quarter of the measured value. The amount of hydroxyl groups on the wood cell surface may influences the interaction of nitrogen with the wooden surface due to the quadrupole moment of nitrogen^57^. This can be one reason for the high deviations in the nitrogen physisorption measurements. Based on the high standard deviation, nitrogen as adsorptive appears to be unsuitable for physisorption analysis of wood. This contradicts hitherto published research where nitrogen physisorption was used to differentiate between species, structural parts or modifications^28,29,56^.

So far, pore size determination on dry wood was performed by applying Barrett-Joyner-Halenda (BJH) method or density functional theory (DFT) to sorption isotherms^18,27,29^. This can only be done when the pore shape is known or can be assessed by other methods^13,58^. Therefore the determination of pore size distribution with nitrogen or krypton physisorption is not possible for wood. The use of mathematical models like density functional theory (DFT) or non-local-density functional theory (NLDFT) for pore size detection is inapplicable to this material type in general.

Krypton as adsorbate for surface area measurements offers a resolution of 0.05 m² ^59^. This can be seen in the present study’s small standard deviation of krypton physisorption. Surface area for krypton is up to 6 times lower than for nitrogen. Similar results for krypton and nitrogen measurements were reported for measurements on membranes^25^. In the study of Orsolini *et al*. (2015) it was concluded that this shows an anomalous effect of nitrogen and therefore the BET model application is questionable. Due to low standard deviations between krypton, even distinctions of earlywood and latewood as well as between sapwood and heartwood of Douglas fir are possible. Earlywood shows higher surface areas compared to latewood due to the greater cell lumina. This is supported by the comparison of microscopic measurements and surface area calculations. It is important to mention that the determination of surface area by krypton physisorption has to be handled carefully due to different possible cross-sectional areas of krypton which vary with the solid^13^.

The microscopic measurements of a predicted surface based on an assumed longitudinal lumen correspond well with the krypton values. Physisorption detects the entire accessible three-dimensional surface area, while the microscopic method is a two-dimensional analysis. This could give rise to errors, but the strong accordance between krypton and microscopic determined surface area, especially in comparison to the nitrogen physisorption values, indicate that for organic materials like wood with very low surface are krypton is a more reliable adsorptive the nitrogen. Scientific literature on nitrogen physisorption on wood should therefore be viewed critically.

### High pressure hydrogen physisorption

**Figure 3:**
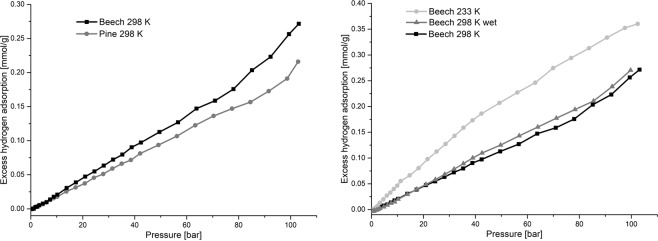
High pressure hydrogen adsorption isotherms (left beech and pine at 298 K, right beech at 233/298 K dry and 298 K with about 8% moisture content).

High pressure measurements with hydrogen were done for beech and pine between 1 and 100 bar and at different temperatures. The results are shown in Fig. 3. Both wood species show no significant absorption of hydrogen in the dry and humid state in the whole pressure region.

Nitrogen and krypton physisorption measurements do not prove that inside the wood cell there are pores or surface areas undetectable by the chosen adsorptive due to their molecular size or pore blocking at dry state. For the first time, high pressure hydrogen sorption measurements were performed on wood. If hydrogen was to enter empty spaces in dry wood a non-linear curve would be observed. The results show linear uptake with no significant additional amount of adsorbed hydrogen at higher pressures. Therefore, we conclude that no accessible pores or no volume in general exist in the cell wall at dry state. A consequence of this is that gaseous molecules in physisorption are only adsorbed on the outer surface of the dry wood cell wall. Hence investigations with these techniques will give no explanation on the structure and porosity within the wood cell wall. The absence of spaces at dry state is in contradiction to a preexisting micro porosity at dry state in the wood cell wall. Further experiments and comparisons should be done to prove the new findings. Douglas fir was investigated via krypton physisorption, because this method gave the most precise results for pine and beech (Table 2) and this species allows precise separation of earlywood and latewood. Measurements were made with heartwood and sapwood as well as latewood and earlywood (Table 3). The results show that surface area is at least 4 times higher for earlywood than for latewood. The sapwood shows lower values in earlywood and latewood compared to heartwood.

### Thermoporosimetry

**Figure 4:**
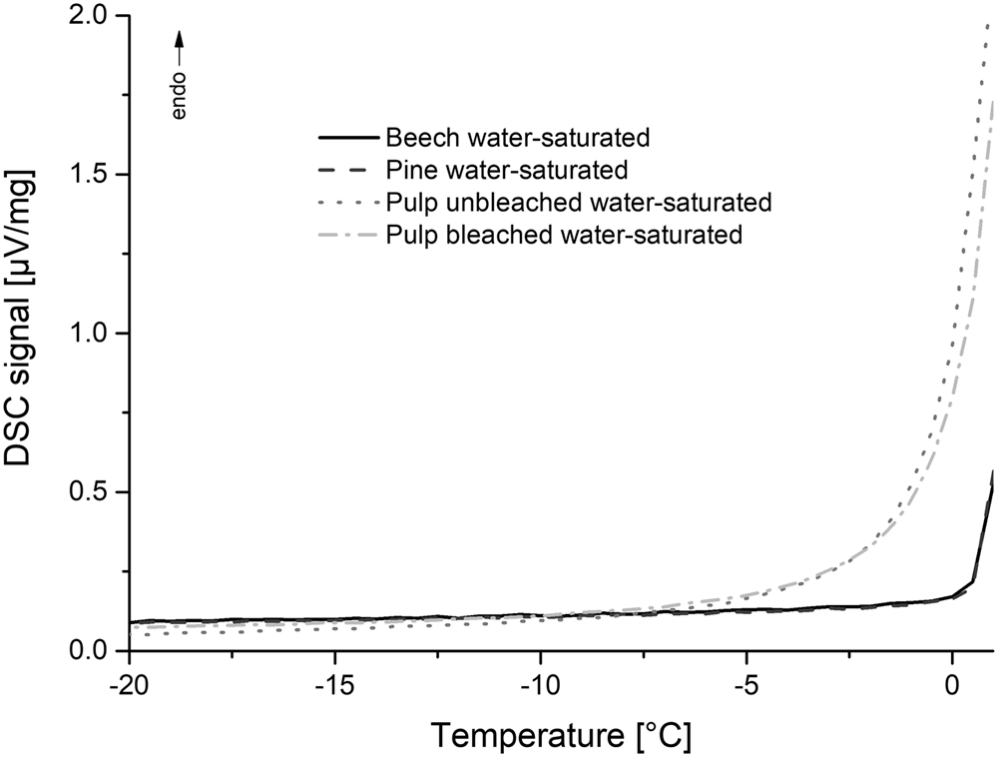
Ice melting with linear heating of 1 K/min of fully saturated wood (beech and pine) and pulp (coniferous wood, bleached and unbleached).

Fully saturated beech and pine wood as well as pulp were measured with a linear heating rate of 1 K/min from −70 °C to 25 °C. The melting behavior is shown in Fig. 4. First derivative of the heating curves indicate a melting for bleached and unbleached pulp at −6 °C and for wood at 0 °C.

**Figure 5:**
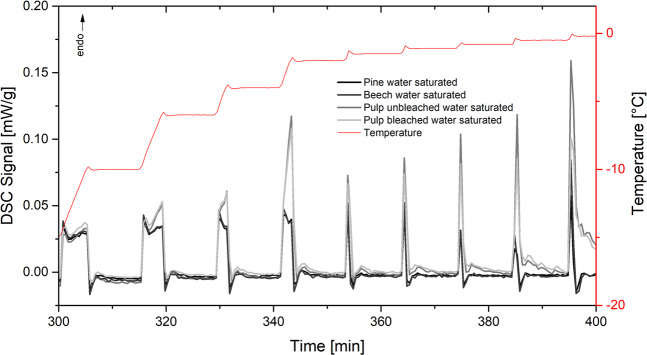
Isothermal step thermoporosimetry of beech/pine and bleached/unbleached pulp, region of interest between −20 and 0 °C, heating rate 1 K/min.

Both wood and pulp samples were also measured in fully water-saturated state with the isothermal step TPM procedure as can be seen in Fig. 5. Also, with this procedure a difference between the melting peaks of wood and pulp can be seen.

**Figure 6:**
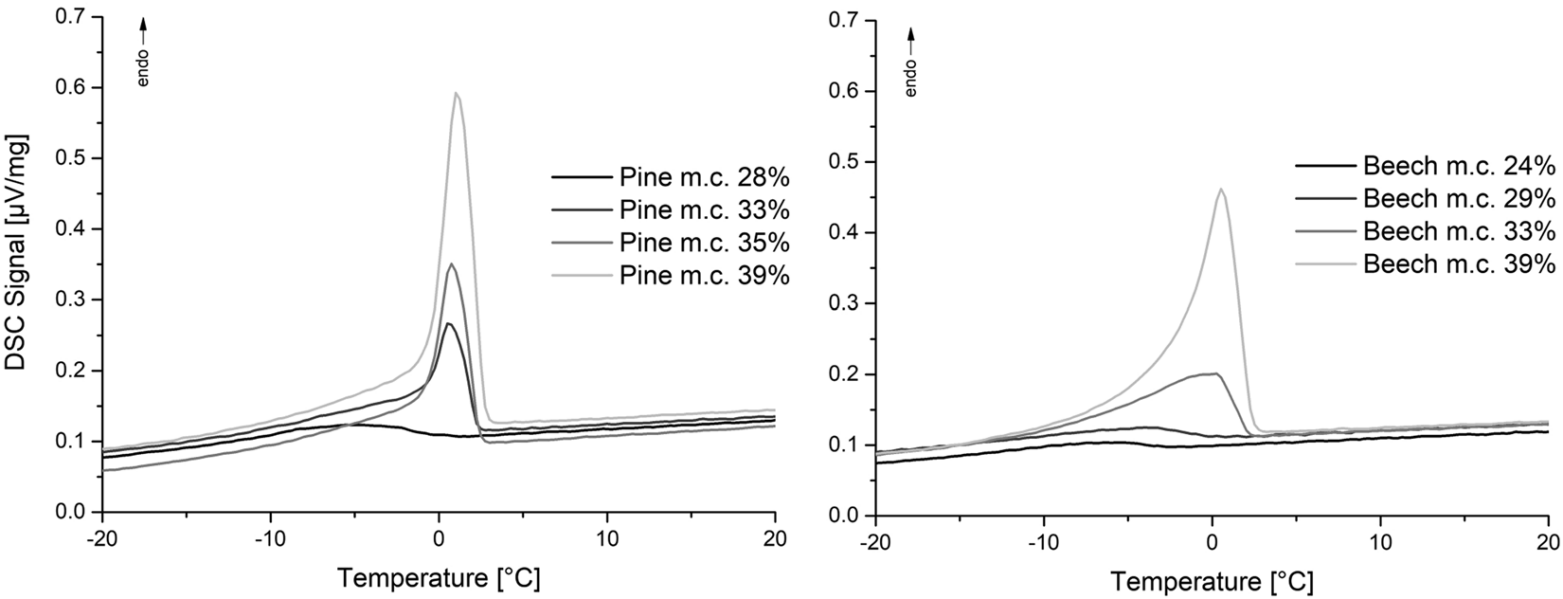
DSC heating curves with heating rate of 1 K/min (left pine, right beech).

Oven-dried wood samples rewetted with different moisture contents were cooled down and heated up in DSC for detecting the freezing point (Fig. 6). The results show that for a moisture content below 28% (pine) and 29% (beech) no significant melting of water can be observed. With higher moisture contents the melting peak is increasing.

**Figure 7:**
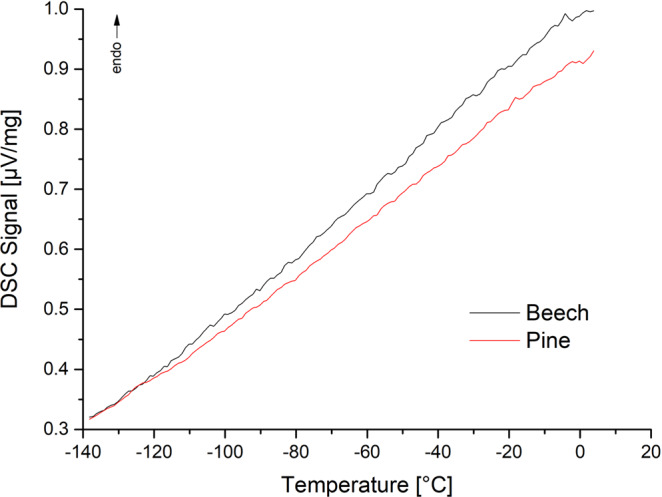
DSC heating curves with heating rate of 1 K/min (left pine with moisture content 18,98%, right beech with moisture content 16,65%) starting from −140 °C indicating no melting behavior even in lower temperature ranges.

In Fig. 7 DSC heating curves of beech and pine starting from lower temperatures are shown. Both wood samples show no melting of water even at low temperatures.

Focussing on the wet state, a difference in the melting behavior close to 0 °C between pulp and wood in fully saturated state can be seen. Melting can be observed for pulp but not for wood (Fig. 4). Thermoporosimetry is therefore more applicable to pulp because pores can detected when lignin is removed and enough space is available to form ice cluster^23^.

In the saturated state, small amounts of water can be detected with the isothermal step method (Fig. 5) which is in accordance to relevant literature^19,55^. Below fiber saturation no melting can be observed (Fig. 6) which supports the assumption that in the cell wall only non-freezing bound water is present^7,60^. This is also proved by the measurements in Fig. 7. Even at temperatures down to −140 °C, no melting can be observed indicating that no freezable water exists in very small mesopores.

At high moisture contents via absorption, melting of water can be observed close to fiber saturation. Recent studies found that fiber saturation is a transition region^61^ with high hysteresis^62^. Between 95% and 100% relative humidity climate water is condensing in biological formations outside the cell wall, like pits, and this water can be detected by low field NMR^63^. These structures are not created by water sorption into the cell, otherwise a higher swelling ratio within this region would have been observed, which is not the case as the swelling is linear^64^. The absence of melting below fiber saturation and the detection of freezing water close to and above this point lead to the conclusion that below fiber saturation no mesopores are present within the cell wall and the hypothesis of water induced meso-porosity in the hygroscopic region is not correct.

The application of the Gibbs-Thomson-equation to biopolymers like wood has to be viewed critically. For pulp, the pore size and melting temperature cannot be easily calculated by the Gibbs-Thomson-equation^48^. The contact angle is assumed to be 180° for organic materials^47^, but a proof for this is challenging due to the unknown behavior of water in the cell wall. Additionally, wood is an amphiphilic material with hydrophilic and hydrophobic parts^65,66^ influencing the contact angle between water and the surface^67^. The assumption of cylindrical pore geometry is also questionable due to the natural structure of the biopolymers. Currently, no technique is available for determining the pore geometry within the wood cell wall. Temperature dependencies of the parameters of the Gibbs-Thomson-equation must be determined or assumed which is more complicated for organic materials than for inorganic ones. Therefore, thermoporosimetry has to be handled as an uncertain method when wood is investigated.

In this paper we have focused on mesopores within the wood cell wall. Beside this pore class also micropores can exist. As they are not detectable with cryo-techniques because they are below 2 nm and therefore show no freezing behavior^37,39^, their existence in the native wood matrix is an open question. Micropores in organic materials are so far difficult to detect by experimental methods. For example Donaldson *et al*.^68^ investigated the porosity of cell wall surfaces in comparison to the internal porosity with molecular microscopy. Also molecular dynamic simulations could help in further understanding of wood-water-interactions by investigating micropores in the hygroscopic region as has been done for micropores in cellulose^21^.

The presented results strongly support the assumption that mesopores are not present within the dry or wet wood cell wall. This is in contradiction to existing literature were mesopores are show for wood with mercury intrusion porosimetry^16^, physisorption^17,18^, thermoporosimetry^19,20^ or by dynamic water vapour sorption measurements^69^. Therefore, the previous discussed information as well as the presented results indicate that the measurement techniques are limited for investigations of mesopores in wood.

When water is held in cell wall structures lower than 2 nm, transport mechanism within the wooden cell wall can be seen from a different perspective. At this level surface diffusion occur as main transport mechanism. Another explanation is the transport of water through rubbery amorphous polymers as shown by Jakes^70^. Generally, these approaches are in contradiction to bulk diffusion assumptions. Therefore the presented research supports the assumption that wood on molecular scale can also be seen as swelling of a gel rather than a solid which was first supposed by Barkas^71^.

## Conclusions

Several characterization techniques were used to investigate the cell wall porosity of wood. The accuracy of nitrogen physisorption is not sufficient to determine the surface area of wood. Determination of pore sizes and pore size distribution with nitrogen are not reliable either. High pressure hydrogen physisorption supports the assumption there are no pores or surfaces within the cell wall substance. Therefore, we conclude that with gas physisorption only the porosity of the cell wall surface can be detected and no penetration of the bulk cell wall material itself takes place. Krypton is a more reliable adsorptive for the determination of small surface areas than nitrogen and thus should be applied to organic materials like wood. The low surface area found by krypton and supported by microscopic comparison calculations raises the question how the cell wall surface is structured. Thermoporosimetry can detect no mesopores in wood which is in contradiction to existing literature. Below equilibrium moisture content there is only non-freezing-bound water, therefore pores detected by thermoporosimetry must have previously existed by biological structures or cavities. Above that threshold, water can condense in major structures and can be detected as it freezes. No water vapor induced mesopores were found within the hygroscopic region. Therefore, we reject the hypothesis that water vapor sorption leads to mesoporous structures within the cell wall. Hardwood and softwood behave similarly indicating that the results are representative for wood in general. Thus, when using the term cell wall porosity one needs to distinguish between pores on the cell wall surface and pores that are existing because of biological structures. The findings offer the possibility to renew basic wood-water-sorption theories. Transport of water in the cell wall must be realized by structures lower than two 2 nm. This is only possible by surface diffusion or by transport through rubbery polymers like gels, which is in contradiction to bulk diffusion assumptions. Also, wood modification, wood degradation and transport processes within the wood cell wall can be seen from a different perspective due to the transport behavior of molecules.

## Material and methods

Never dried bleached and unbleached softwood sulfite pulp was used. Industrially dried beech (*Fagus sylvatica*) and Scots pine (*Pinus sylvestris*) were used. Air dried Douglas fir (*Pseudotsuga menziesii*) was used. Earlywood and latewood was separated by a broach. Transition regions between earlywood and latewood were cut out.

### Nitrogen/krypton physisorption

A Masterprep degasser (Quantachrome Instruments) was used for sample preparation. Samples were dried prior to measurements at 30 °C in rotary vacuum pump for 48 hours. Douglas fir heartwood and sapwood slices of 1.5 mm thickness in longitudinal direction were cut with a bench saw. Afterwards earlywood and latewood were separated manually with a broach.

Low pressure physisorption measurements using nitrogen and krypton as adsorbates were performed using a Quadrasorb evo MP by Quantachrome Instruments. Adsorbates were used in high purities (5.0 for nitrogen and 6.0 for krypton).

Measurements and data evaluation were made in software QuadraWin (Quantachrome Instruments). The calculation of the surface area was carried out following the theory of Brunauer, Emmet and Teller (BET)^72^ as recommended by the IUPAC^13^.

### Biological microscopy

A radial bar of Douglas fir was cut with a bench saw. From sapwood, cubic pieces with an edge length of 1 cm were cut with a handsaw. Afterwards the samples were immersed in water and then cut with a microtome to the thickness of 10 µm. Then the samples were immersed into sodium hypochlorite (2.8% from DanKlorix) for 15 minutes and afterwards rinsed with water. After that the samples were dyed with safranin for 10 minutes and then rinsed with ethanol. Then all samples were embedding into Euparal and then dried at 60 °C for 10 days in a drying oven.

Images were recorded with a digital microscope VHX-5000 from KEYENCE, Japan and a digital camera VHX-5020. The image evaluation was performed with the VHX-5000 Software. For the brightness threshold the difference between cell walls and cell lumina was set. Afterwards a manual posttreatment of the automatized image evaluation was done to correct false software detections. Image evaluation was performed at three different local points within two different annual rings for earlywood and latewood. In total 5.8 mm² with 4222 cells in earlywood and 4.8 mm² with 7375 cells in latewood were detected.

The detected surface areas and circumference from microscopy were used to calculate a theoretical value of surface area per gram. Based on the bounded measured area surfaces for cell wall and lumina were calculated. The surface area of the cell lumina was computed by the assumption of a 1000 µm long lumen with the radius of the measured microscopic value. For the sample weight the cell wall substance of the measured sample was calculated by the assumption of a 1000 µm long sample and a cell wall density of 1.5 g/cm³.

### High pressure hydrogen physisorption

Beech and pine wood samples with dimensions of approximately 10 × 4 × 4 mm where used. The volumetric high pressure hydrogen adsorption measurements were carried out on a BELSORP HP high pressure gas adsorption measuring system in combination with a Julabo deep-freeze circulating thermostat FP89 ME in a pressure range up to 110 bar. The same combination was used for the determination of the bulk-density of wood with helium at 103 °C for 24 h with an oil vacuum pump.

The adsorption experiments do not determine the total amount of adsorbed gas, but only the excess adsorption, which is caused by a density increase of the adsorbing species in the volume of the adsorption layer due to the interaction with the solid. The relationship between the total adsorbed gas and excess adsorption can be described by the Eq. 1:

1 $${N}_{ex}=\,{N}_{abs}-{\rm{\rho }}\cdot {\rm{V}}$$

With the excess adsorption amount *N*_*ex*_, the total adsorbed amount of gas *N*_*abs*_, density of the gas in the volume phase *ρ and the* pore volume V^73^.

The determination of the volumetrically measured values *N*_*ex*_ and *N*_*abs*_ are based on standard temperature and pressure, so that the adsorbed volume is converted into the mass of the adsorbed gas and the storage capacity is given as a percentage of the total wood mass.

### Thermoporosimetry

All TPM measurements were performed twice. Wood slices with dimension 1.5 × 10 × 10 mm were cut with a bench saw. Afterwards round slices with thickness of 1.5 mm and diameter of 4 mm were cut with a hole punch. All fully saturated samples were immersed in water prior to the measurements for at least 3 days. Dry samples were stored in an oven at 103 °C for 48 hours. Before the determination of melting area, the wood samples were dried for 48 hours at 103 °C in an oven, followed by a partial rewetting with water. Samples and the required amount of water were placed into the DSC pan and the pan was closed. Afterwards the pan with water and sample was stored for five days prior to the measurements.

For TPM measurements a Netzsch DSC 204 F1 Phoenix (heat flux differential scanning calorimeter) was used with a cooling ring for the connection to an intracooler (T → −70 °C), a cooling port for liquid nitrogen (T → −180 °C). Hermetically sealed 30 μl aluminum concave pans were used. Purge gas was nitrogen with flow rate of 40 mL/min.

The temperature values and the heat flow were calibrated in a range of −190 to 600 °C with adamantane, indium, bismuth, tin, zinc, cesium chloride (calibration kit from Netzsch), ultrapure water and potassium nitrate (Merck 105065) according to the specified heating rates.

NETZSCH Proteus – Thermal Analysis – Version 7.1.0 was used for data evaluation. For the TPM measurements the samples were cooled down to −100 °C and heated up stepwise (Table 1). The enthalpy of each step was calculated via integrating each endothermal peak with left starting horizontal baseline.

A reduced Gibbs-Thomson-equation is used for lignocellulosic materials^45,47,50^ where the contact angle is assumed to be 180° (Eq. 2). Also, that the wood does not dissolve in water as well as cylindrical pore shape is assumed. The heating rate within is these measurements was set to 1 K/min.

2 $$D=\frac{-4{T}_{0}\,\gamma \,\cos \,{\rm{\theta }}\,}{({T}_{0}-{T}_{m})\,\rho {H}_{f}}$$

With and average pore diameter *D* (m), melting temperature of pure water T_0_ (K), melting temperature of water in voids T_m_ (K), contact angle between water and ice and wood θ, surface energy γ (J m^−2^), density of water ρ (g m^−3^) and specific heat of fusion H_f_ (J g^−1^).

## Data Availability

The datasets generated during and/or analysed during the current study are available from the corresponding author on reasonable request^74^.
